# Mouse Lines with Cre-Mediated Recombination in Retinal Amacrine Cells

**DOI:** 10.1523/ENEURO.0255-21.2021

**Published:** 2022-02-11

**Authors:** Didem Göz Aytürk, Wenjia You, Constance L. Cepko

**Affiliations:** 1 Department of Genetics, Harvard Medical School, Boston, MA 02215; 2 Howard Hughes Medical Institute, Chevy Chase, MD 20815; 3 Department of Genetics and Ophthalmology, Blavatnik Institute, Harvard Medical School, Boston, MA 02215

**Keywords:** amacrine, Cre recombinase, fluorescent reporter, inhibitory interneurons, mouse genetics, retina

## Abstract

Amacrine cells (ACs) are the most diverse neuronal cell type in the vertebrate retina. Yet little is known about the contribution of ACs to visual processing and retinal disease. A major challenge in evaluating AC function is genetic accessibility. A classic tool of mouse genetics, Cre-mediated recombination, can provide such access. We have screened existing genetically-modified mouse strains and identified multiple candidates that express Cre-recombinase in subsets of retinal ACs. The Cre-expressing mice were crossed to fluorescent-reporter mice to assay Cre expression. In addition, a Cre-dependent fluorescent reporter plasmid was electroporated into the subretinal space of Cre strains. Herein, we report three mouse lines (*Tac1::IRES-cre*, *Camk2a-cre*, and *Scx-cre*) that express Cre recombinase in sub-populations of ACs. In two of these lines, recombination occurred in multiple AC types and a small number of other retinal cell types, while recombination in the *Camk2a-cre* line appears specific to a morphologically distinct AC. We anticipate that these characterized mouse lines will be valuable tools to the community of researchers who study retinal biology and disease.

## Significance Statement

Amacrine cells (ACs) are highly diverse, inhibitory interneurons of the retina. The roles of the majority of these ACs are poorly understood, particularly in terms of their role in visual processing and retinal disease. The present study describes three mouse strains with Cre-recombinase expression in specific subpopulations of ACs. As Cre-loxP constructs allow efficient labeling and manipulation of cells *in vivo*, we anticipate that the mouse models will be valuable tools in the study of AC biology.

## Introduction

The mammalian retina is a highly organized neuronal tissue lining the back of the eye (Rodieck and Rodieck, 1998). Five major classes of retinal cell types are organized into complex circuits that are responsible for extracting valuable features from the visual scene, such as color, contrast, luminance and motion (Masland and Raviola, 2000; Gollisch and Meister, 2010). Signal detection and transduction originate in photoreceptors (PRs), which use the rate of release of glutamate to signal light level information to bipolar (BP) neurons. Most BP cells, which are also glutamatergic, signal to the output neurons of the retina, the retinal ganglion cells (RGCs). Rod BPs constitute a separate major pathway where they route the rod PR signal to AII amacrine cells (ACs) that in turn connect with RGCs and cone BP cells. RGCs relay these signals to specialized retinorecipient areas in the brain. Inhibitory neurons are critical to the proper information transformations conducted by the many types of circuits. These inhibitory neurons are ACs and horizontal cells (HCs), with ACs being >10-fold more abundant than HCs. GABA and glycine are the principal neurotransmitters used by ACs, which can also use other neurotransmitters, such as dopamine and serotonin, as well as a variety of neuropeptides. The contribution of ACs to the complexity of retinal processing is also indicated by their diversity. Single-cell RNA sequencing (scRNA-seq), as well as more classical anatomic and physiological descriptions, have defined at least 63 types of ACs in the mammalian retina (Helmstaedter et al., 2013; Macosko et al., 2015; Yan et al., 2020).

The cell bodies of ACs, BPs, and HCs reside in the inner nuclear layer (INL) of the retina, with ACs comprising ≈41% of all cells in this cellular layer (Strettoi and Masland, 1995; Jeon et al., 1998). ACs send their processes to the inner plexiform layer (IPL), where they connect to BPs and RGCs via chemical and/or electrical synapses. This very rich synaptic layer is where most of the information transformations occur. The IPL is divided into two main subdivisions, according to the responses that the RGCs make on light stimulation. When RGCs fire with the onset of light, their connections tend to be in sublamina *b*, the inner half of the IPL, the ON layer. Conversely, those that fire when light goes off target sublamina *a*, the outer half of the IPL, the OFF layer (Morgan and Wong, 1995). The separation of ON and OFF pathways is an important distinction that contributes to our understanding of the role played by various cell types. Defining stratification patterns of the interneurons in the IPL thus provides critical clues as to the types of circuits a cell type might contribute to.

ACs are responsible for a variety of inhibitory and neuromodulatory functions in visual processing (Witkovsky, 2004; Demb and Singer, 2012; Taylor and Smith, 2012). For example, AII ACs, the most abundant AC type in the mouse retina, transfer rod BP cell (BPC) signals to cone BPCs, thereby contributing to scotopic vision (Demb and Singer, 2012). Cholinergic, wavelike signaling from starburst ACs in early development is indispensable for the proper formation of visual system circuitry (Ford et al., 2012). Starburst ACs are also critical in the detection of motion in a particular direction (Euler et al., 2002). In addition, there is evidence indicating that ACs contribute to the adaptation of retinal circuitry to bright light, by setting the gain under strong illumination, as well as synthesizing nitric oxide (NO) and thereby playing a role in the vasomotor responses of the retina (Jacoby et al., 2018). More recently, a role was suggested for ACs in the sensitization of RGCs, by elevating local sensitivity in the retina following a strong visual stimulation (Kastner et al., 2019).

The aforementioned functional needs are thought to be met by the tremendous diversity of ACs in terms of their morphology and genetic profiles, as well as in the modes they use for communicating with other cells in the retina. Despite our appreciation of the importance of ACs in the mammalian retina, we are far from assigning functional or disease-relevant roles to the majority of AC types. This is in part because of lack of genetic handles that would enable labeling or manipulating specific AC types. Our goal in the current study was to establish genetic tools for studying AC function in the retina. A classic tool enabling selective manipulation of specific cell types is Cre-mediated or Flp-mediated recombination, achieved by crossing Cre-expressing or Flp-expressing mice to recombinase-sensitive strains (Dymecki et al., 2010). Recombinase expression can be driven by promoters of choice from specific loci in the mouse genome. With the morphologic and functional AC diversity in mind, we set out to examine existing strains of mice that express Cre in subsets of neurons. We compiled a list of candidate Cre-expressing mouse lines by reviewing a single-cell gene expression database (Trimarchi et al., 2007; Cherry et al., 2009) as well as published reports of marker genes expressed by AC subsets (e.g., particular neuropeptides). We then bred selected Cre lines to fluorescent protein Cre-reporter mice and used electroporation of Cre-dependent plasmids. Here, we report three mouse strains that we identified as of potential interest to the community who study the retina, with a particular focus on AC types.

## Materials and Methods

### Mouse lines

All animal procedures were conducted in accordance with Harvard Medical School guidelines and approved by IACUC at Harvard University. Genetically modified mouse strains with Cre expression were obtained from the resources listed in Table 1. *Scx-cre* was a kind gift from the Tabin laboratory at Harvard Medical School.

**Table 1:** Cre expressing mouse lines used in the AC screen

Cre mouse line	Gene/gene family	Gene insertion method	Source
*Tac1::IRES-cre* *[Tac1-IRES2-Cre-D]*	Tachykinin precursor 1/tachykinin peptide hormone family	Insertion into the locus	Jackson (stock #021877); Harris et al. (2014)
*Camk2a-cre* *[Tg(Camk2a-cre)]*	Calcium/calmodulin-dependent protein kinase II Alpha/serine/threonine protein kinases family	Transgenic	Jackson (stock #005359)
*Scx-cre* *[Tg(Scx-Cre)]*	Scx/bHLH family	Transgenic	MGI:5317938; Blitz et al. (2009)

All mice were crossed to a Cre recombinase-dependent reporter mouse line that expresses tdTomato from the ROSA26 locus (Ai9, JAX #007909). Mice of either sex were used in all experiments.

### Immunohistochemistry (IHC)

Adult mice were euthanized with CO_2_ and decapitation, and their eyes were enucleated. Corneas and lenses were removed, and retinae dissected out in cold PBS. Freshly dissected retinae were fixed in ice cold 4% formaldehyde for 30 min before 3× wash with PBS. Retinae were switched to a sucrose gradient for cryoprotection and kept in 30% sucrose in PBS at 4°C until they sank. Sections were cut in OCT at 20–25 µm with a Leica CM3050S cryostat (Leica Microsystems) and preserved at −80°C until further use.

For immunostaining, sections were removed from the freezer and acclimated to room temperature. After 3× wash with PBS, sections were blocked in 6% donkey serum in PBS with 0.3% Triton X-100 (blocking solution) for at least 30 min. Primary antibody in blocking solution was applied to the slides and incubated overnight in the cold room at 4°C in a humidified chamber (see below for detailed information on the primary antibodies used and the dilutions). The following day, sections were washed 3× with PBS, and secondary antibody in blocking solution was applied to the slides for 3 h at room temperature (below for detailed information on the secondary antibodies used). Afterwards, the slides were washed again 3× with PBS (second wash included a DAPI co-stain). Finally, all slides were mounted in Fluoromount-G (Southern Biotech).

Primary antibody dilutions used in this study were as follows (Table 2): goat anti-choline acetyltransferase (Millipore; AB144P, 1:30), goat anti-Brn3a (Santa Cruz, sc-6026, 1:400), rabbit anti-GABA (Sigma-Aldrich, A2052, 1:500), goat anti-GlyT1 (Chemicon, AB1770, 1:2500), rabbit anti-TH (Millipore Sigma, AB152, 1:1000), rabbit anti-Calbindin (Swant, CB38, 1:2000), rabbit anti-Dab1 (Millipore Sigma, AB5840, 1:250), mouse anti-Satb2 (Abcam, ab51502, 1:25), guinea pig anti-VGlut3 (Millipore Sigma, AB5421, 1:1000), and rabbit anti-RFP (Abcam, AB62341, 1:1000).

**Table 2:** Primary antibodies used in the study

Antibody	Retina labeling	Raised in/clonality	Source
Anti-ChAT	Starburst ACs	Goat/polyclonal	Millipore Sigma, AB144P
Anti-BRN3a	RGCs	Goat/polyclonal	Santa Cruz, sc-6026
Anti-GABA	GABAergic ACs	Rabbit/polyclonal	Millipore Sigma, A2052
Anti-GLYT1	Glycinergic ACs	Goat/polyclonal	Chemicon, AB1770
Anti-TH	Dopaminergic ACs	Rabbit/polyclonal	Millipore Sigma, AB152
Anti-CALBINDIN	Subset of ACs and HCs	Rabbit/polyclonal	Swant, CB38
Anti-DAB1	AII ACs	Rabbit/polyclonal	Millipore Sigma, AB5840
Anti-SATB2	nGnG ACs and some RGCs	Mouse/monoclonal	Abcam, ab51502
Anti-VGLUT3	Glutamatergic ACS	Guinea pig/polyclonal	Millipore Sigma, AB5421

Choline acetyltransferase (ChAT), brain-specific homeobox/POU domain protein 3A (BRN3a), Gamma-aminobutyric acid (GABA), Glycine transporter type 1 (GLYT1), Tyrosine hydroxylase (TH), Disabled-1 (DAB1), Special AT-rich sequence-binding protein 2 (SATB2), Vesicular Glutamate Transporter 3 (VGLUT3).

Secondary antibodies and dilutions used in this study were as follows: donkey anti-goat Alexa Fluor 647 (1:250, Jackson ImmunoResearch), donkey anti-goat DyLight 649 (1:250–1:400, Jackson ImmunoResearch), donkey anti-rabbit Alexa Fluor 647 (1:250, Jackson ImmunoResearch), donkey anti-rabbit DyLight 649 (1:250–1:400, Jackson ImmunoResearch), donkey anti-mouse DyLight 649 (1:250–1:400, Jackson ImmunoResearch), donkey anti-mouse DyLight 488 (1:250–1:400, Jackson ImmunoResearch), and donkey anti-guinea pig FITC (1:400, Jackson ImmunoResearch).

### *In vivo* electroporation and plasmid delivery

*In vivo* electroporation was used to deliver plasmids into the subretinal space, as previously described (Matsuda and Cepko, 2007; updated electroporation protocol, described in detail in Wang et al., 2014). Camk2a-Cre pups were injected with a Cre-dependent reporter plasmid (at 1 µg/µl concentration, 0.3–0.5 µl of solution/injection area with two distinct electroporation patches per eye) expressing mCherry (pAAV-flexTC66T; Miyamichi et al., 2013) on postnatal day (p)0. Injections were performed using a pulled angled glass pipette controlled by a Femtojet Express pressure injector (Eppendorf, E5242956003) into the right eye. A short electric pulse was given to the animals right after the injections, to electroporate the construct. After weaning, mice were killed, and the retinas were processed for cryosections, as described above.

### Imaging

Retinal sections were imaged using an inverted Zeiss LSM780 microscope (Carl Zeiss AG) with 405, 488, 561, and 633 nm lasers and a 10× objective (EC Plan Neo Fluor 10×/0.3 Air DIC I) or a 40× oil objective (Plan Apo 40×/1.3 Oil) with Z-stacks. Images were later analyzed with lmaris or ImageJ/Fiji as maximum intensity projections of the image stacks with brightness and contrast adjustments. Channels were overlaid wherever appropriate.

## Results

A number of genetically altered mouse strains with Cre expression driven by selected loci were assayed for expression history within retinal cell types. These mouse lines were either available through public repositories, such as Jackson Labs (https://www.jax.org), or were directly retrieved from research laboratories based on publications. The candidate lines were chosen using at least one of the following criteria or a combination thereof: established expression of a Cre driver gene in a subset of ACs in the literature, expression in ACs according to a retinal single-cell gene expression database (Cherry et al., 2009), and/or specific expression in a subset of inhibitory interneurons in the brain. Knock-in mouse lines were preferred whenever possible, because of a presumed greater fidelity of expression. A few of the Cre lines that we screened were very broadly expressed, or did not express in the retina, or were published for their retinal expression patterns while this work was in progress. These lines are included in Table 3.

**Table 3:** Summary of additional mouse lines that were screened for Cre-mediated recombination in the retina

Mouse line	Retinal Cre-mediated recombination	Existing literature
*Crh-ires-Cre (knock-in, JAX #012704)*	tdTom expression strong in a small subset of ACs in INL, displaced ACs, and RGCs	Similar recombination patterns reported previously in the mouse retina (Zhu et al., 2014; Park et al., 2018)
*Crh-cre (transgenic, MMRRC 030850-UCD)*	tdTom expression strong in a small subset of ACs in INL and a few displaced ACs	No previous reports in the mouse retina; reported in the brain (Niederkofler et al., 2016)
*VIP-cre (JAX #010908)*	tdTom expression strong in a small subset of ACs	Similar recombination patterns reported previously in the mouse retina (Zhu et al., 2014; Akrouh and Kerschensteiner, 2015; Park et al., 2015; Perez de Sevilla Müller et al., 2019)
*NEX-cre (a gift from K. Nave)*	tdTom expression strong in a subset of ACs	Similar recombination patterns reported previously in the mouse retina (Goebbels et al., 2006; Kay et al., 2011)
*Pomc-cre (JAX #010714)*	tdTom expression strong in patches of Mueller glia and some unidentified inner retinal neurons, including HCs	Previous report had a brief statement of no expression in retina; different methodology used (injection of AAV encoding Cre-dependent reporter; Martersteck et al., 2017)
*Sst-cre/ERT2 (JAX #010708)*	Very weak tdTom expression in Mueller glia	No previous reports in the mouse retina; low efficiency recombination reported in the cerebral cortex (Taniguchi et al., 2011)
*Mnx1-cre (JAX #006600)*	tdTom expression dense and strong in Mueller glia and in some unidentified inner retinal neurons	Similar recombination patterns reported previously in the mouse retina (Ivanova et al., 2010)
*Oxy-cre (JAX #024234)*	Very weak tdTom expression in Mueller glia and a few other inner retinal neurons	Brief mention in the mouse retina, expression in ACs, BCs, and HCs; different methodology (injection of AAV encoding Cre-dependent reporter; Martersteck et al., 2017)

All mice were crossed to a Cre recombinase-dependent reporter mouse line that expresses tdTomato (Ai9, JAX #007909), and adult mouse retinal sections were prepared/imaged as described in Materials and Methods.

**Figure 1.**
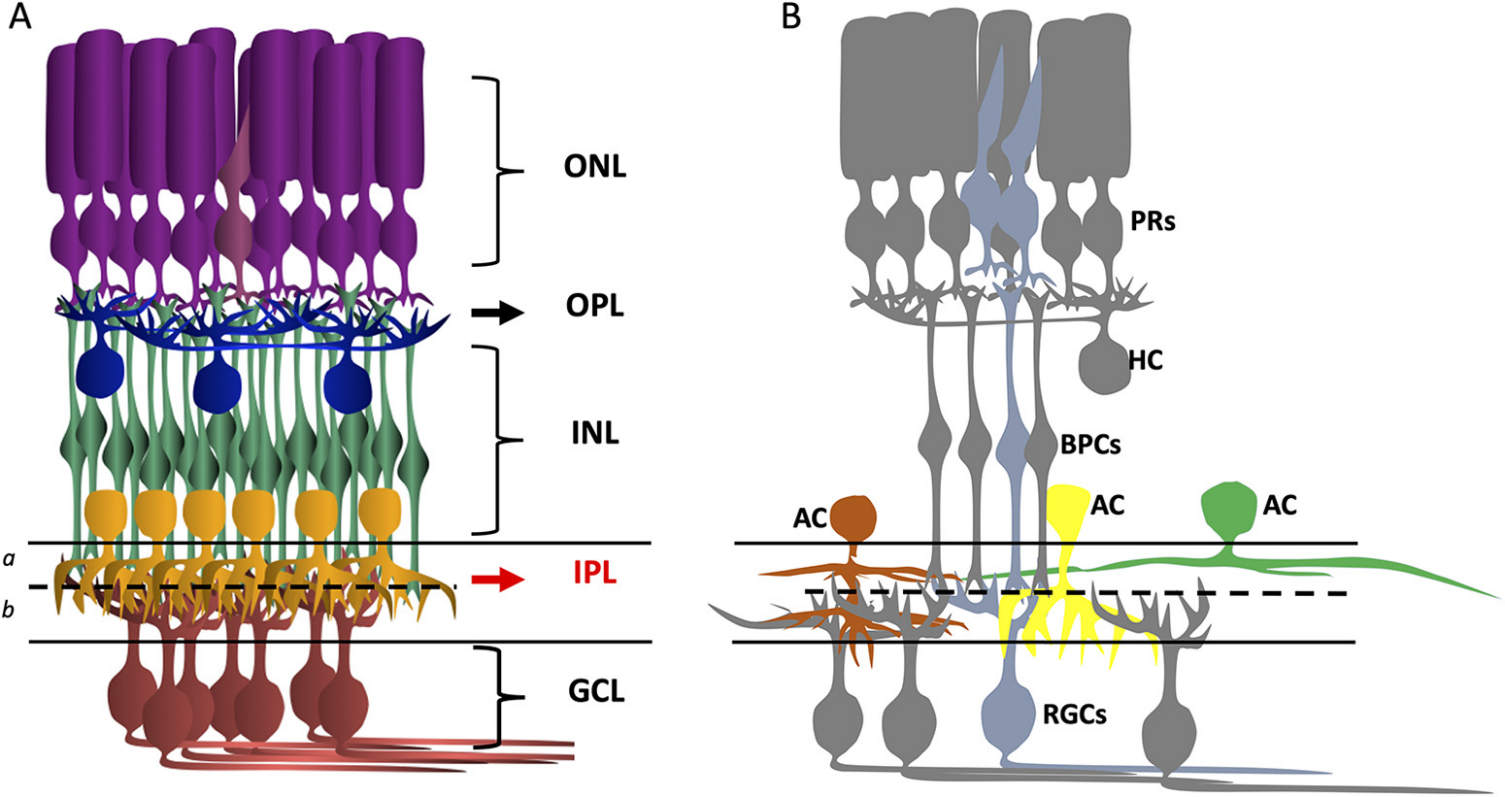
***A***, Schematic of the mouse retina marking the cell layers. IPL divided into sublaminae *a* and *b*. ONL, outer nuclear layer; OPL, outer plexiform layer; INL, inner nuclear layer; IPL, inner plexiform layer; GCL, ganglion cell layer. Dark purple, rods; light purple, cones; blue, HCs; green, BP cells; yellow, ACs; red, RGCs. ***B***, A typical cone circuit has been simplified and highlighted in light blue. ACs with various dendritic shapes, sizes and innervation depths in IPL modify the light signal.

In order to examine the history of Cre activity in mouse lines of interest, Cre mice were crossed to the Ai9 Cre-dependent tdTomato reporter mice, which resulted in bright labeling of subsets of neurons and glia in retinal sections. In Figure 1*A*, different retinal layers indicate landmarks for cell body positions and broad classification of cell types. In Figure 1*B*, a typical cone PR circuit is depicted in blue, where the BPs directly convey the light signal to RGCs. ACs of different arbor sizes and shapes (depicted in orange, yellow and green) extend their processes into different depths of the IPL. Depending on their various morphologic attributes, ACs serve as inhibitory modulators of the light information at multiple levels of the retinal circuitry.

### Cre expression history in retinae of three mouse lines

**Figure 2.**
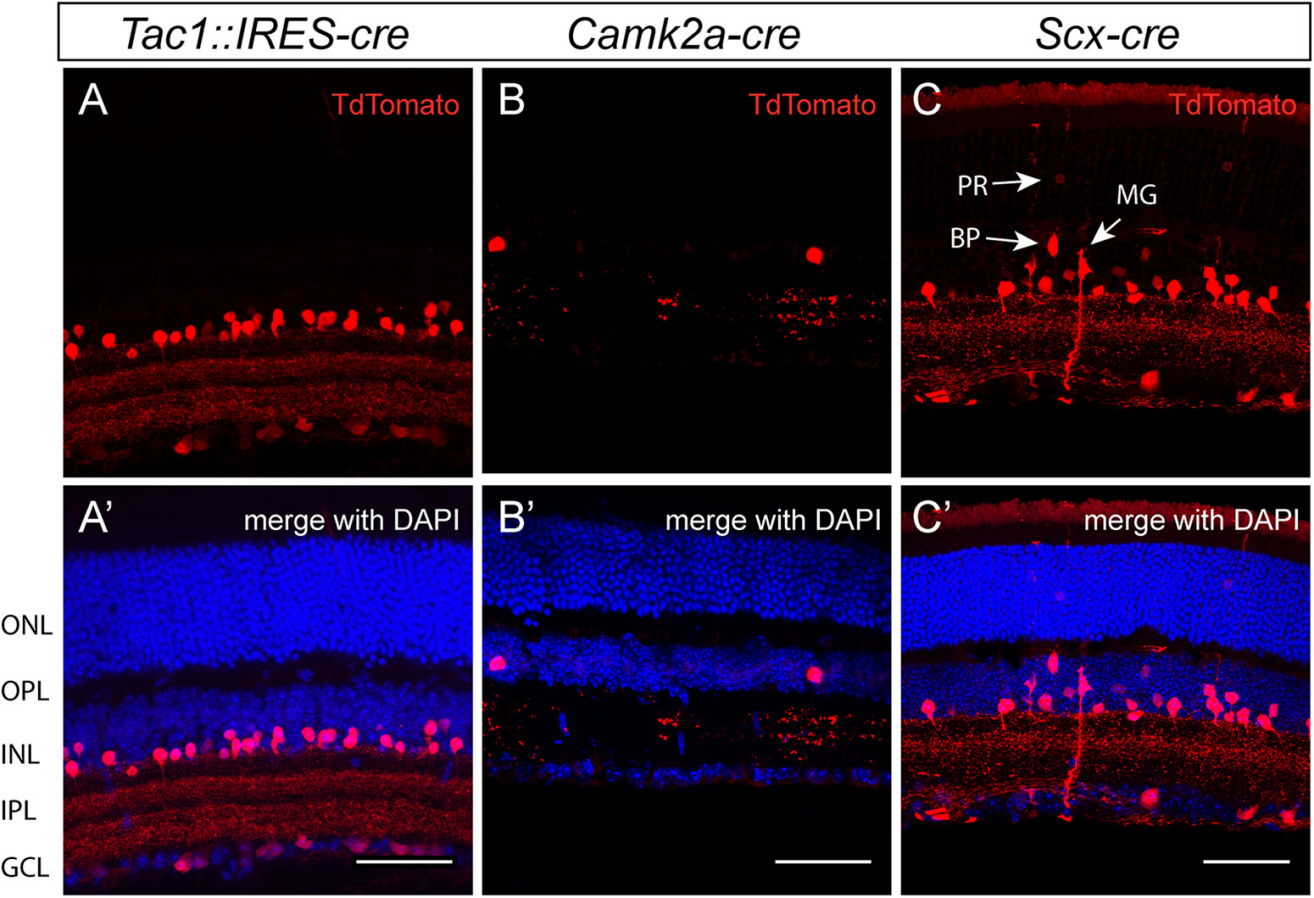
Mouse lines with Cre-mediated recombination in retinal ACs. Three mouse lines with Cre recombinase expression driven by different promoters were crossed to the Ai9 Cre-sensitive reporter line. Retinas were harvested >p21, processed and sectioned to reveal Cre history via TdTomato (red) expression. Cell nuclei were labeled with DAPI (blue). ***A***, Retina of the *Tac1::IRES-cre* mouse has Cre history in a subset of ACs and cells in the GCL that stratify in three distinct depths of the IPL. ***B***, The *Camk2a-cre* mouse line labeled a small number of ACs. ***C***, *Scx-cre* line showed recombination in a subset of ACs, as well as sparsely in the other three retinal cell types generated postnatally [rod (PR), BP, and Mueller glial (MG) cells, indicated with arrows]. Cre recombination was also observed in a small number of cells in the GCL. White scale bars: 50 μm.

Retinae of three different Cre lines crossed to tdTomato reporters were studied for Cre expression history (Table 1). The initial examination of tdTomato expression, and DAPI counterstaining to mark the different retinal layers, showed that all three of the mouse lines had Cre-mediated recombination in subsets of ACs. Some additional cell types were observed in the *Scx-cre* line (Fig. 2). However, all three mouse lines required further studies to determine whether the labeled ACs were a single morphologic/functional type.

In order to assess the identity of cells marked by Cre-mediated recombination, we used frozen retinal sections obtained from Cre lines crossed to the tdTomato reporter mouse, in combination with IHC for various markers of ACs and other retinal cell types. These included antibodies that mark cell identity (such as BRN3A for RGCs and TH for dopaminergic ACs), neurotransmitter content (GABA and GlyT1), IPL stratification patterns and/or identity, such as ChAT (for starburst ACs) and calbindin (refer to Table 2 for a full list of antibody sources).

### The *Tac1-Cre* mouse line labels a distinct subset of GABAergic ACs

*Tac1* encodes a prepropeptide for substance P and neurokinin A. Our previous work using scRNA profiling and gene perturbation studies suggested that *Tac1* is expressed by a small number of ACs (Cherry et al., 2009, 2011). Evaluation of DAPI counterstaining of *Tac1::IRES-cre-tdTom* retinal sections revealed the presence of cells with Cre expression history in the INL and GCL (Fig. 2*A*). There were multiple bright red cell bodies in the inner part of the INL where AC bodies reside, and fewer, dim red cell bodies in the GCL. We could not tease apart the morphologic features of individual neurons in these sections because of high cellular density. However, the labeling pattern resembled the narrow lamination patterns of medium or wide-field ACs, rather than the typical diffuse, multi-layer lamination of narrow field ACs. Three distinct layers of tdTom+ processes in the IPL were detected: one thin stratum in the outer most part of the IPL, right below the INL, a slightly broader stratum in the outer part of the IPL, and an even wider one in the innermost part of the IPL.

**Figure 3.**
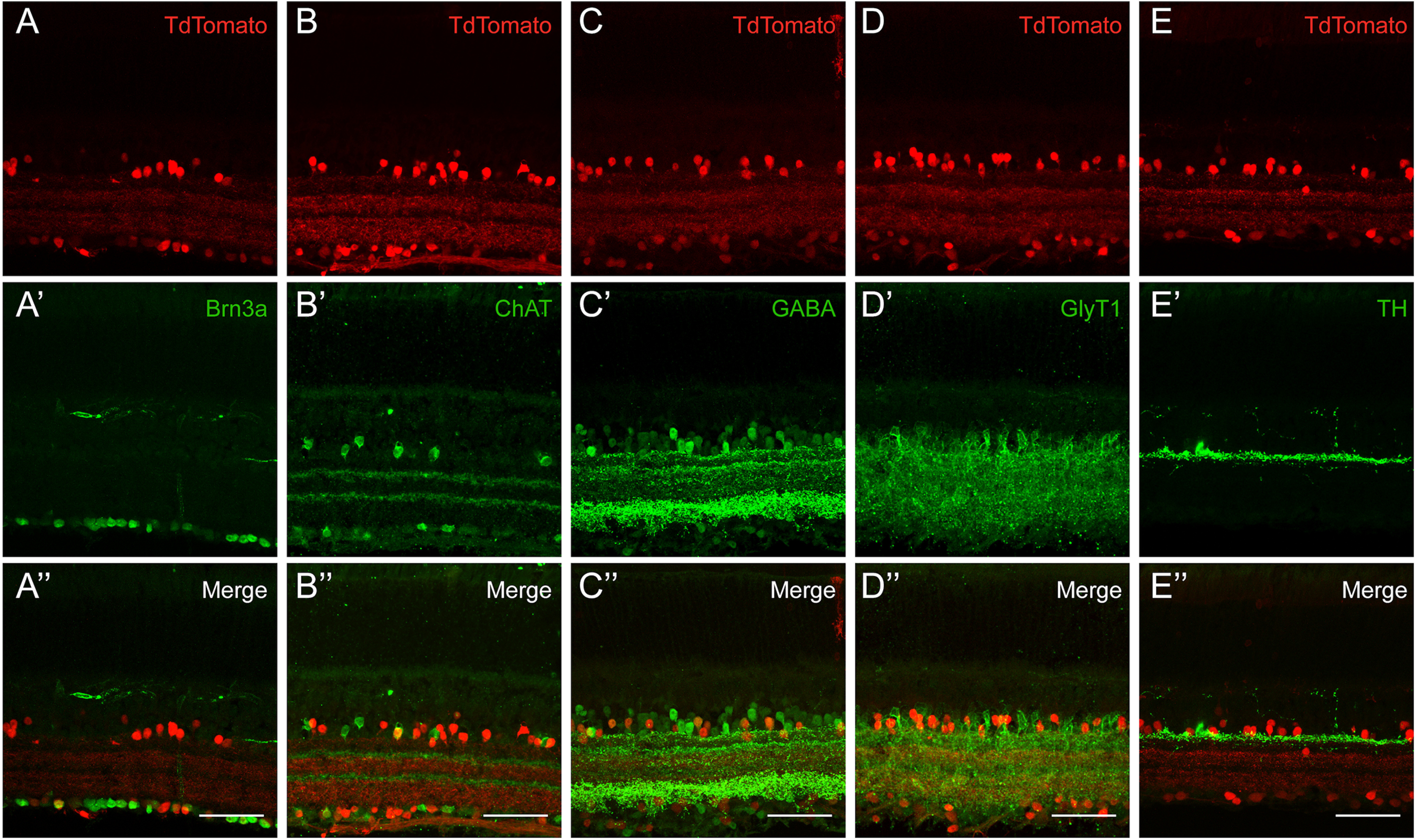
ACs and a small number of RGCs Cre expression history form three unique bands in the IPL of the *Tac1::IRES-cre* mouse. ***A***, Cre expression history in the *Tac1::IRES-cre* mouse could be observed in a subset of ACs and a small number of potential RGCs (Brn3a+ yellow cells in the RGC layer). The presence of red only cells in the RGC layer indicates Tac1-Cre expression history in displaced ACs. ***B***, The distinct stratification pattern of tdTom+ processes in the IPL supports a few morphologically distinct types of ACs being labeled in the Tac1-Cre line. There were no ChAT+ Tac1-Cre cells (the apparently yellow cell in ***B’’*** is because of superposition of 2 cells). The ChAT bands were highly complementary to the Tac1 bands in the IPL. ***C***, ***D***, A majority of the ACs with a Tac1-Cre history were GABAergic, not glycinergic. ***E***, Tac1-Cre cells were not TH-positive. White scale bars: 50 μm.

The GCL of the mouse retina consists of both RGC and displaced AC cell bodies, but there were no clear red axons present in the nerve fiber layer, where RGC axons are found, in the *Tac1* reporter retina. To further examine the identity of the tdTom+ cells in the GCL, sections were stained with an antibody that recognizes BRN3A, a marker of RGCs. A very small number of BRN3A-positive (green) cell bodies with *Tac1-Cre* expression history (red) were observed, suggesting that some of the cells in the GCL might be RGCs (Fig. 3*A*,*A’*,*A’’*, yellow). However, because of the lack of red axons in the fiber layer, and the observed expression of BRN3A in ACs in Brn3 reporter mice, it is possible that these Brn3a+ cells are displaced ACs (Xiang et al., 1996).

The majority of cells in the GCL, however, were only tdTom+, indicating the presence of displaced ACs in *Tac1::IRES-cre* mice. ChAT-positive starburst ACs (green) did not appear to have a *Tac1::IRES-cre* expression history (Fig. 3*B*,*B’*,*B’’*). Interestingly, the stratification patterns in the IPL for starburst ACs and *Tac1* processes appeared complementary to each other, and spanned the entire depth of the IPL. In terms of their neurotransmitter content, *Tac1::IRES-cre-tdTom* ACs (referred to as Tac1-ACs from here on) were found to mostly express GABA (Fig. 3*C,C’*,*C’’*, yellow cells), but there were a few tdTom+ cells that expressed the glycine transporter-1 (GlyT1), the glycinergic marker in the mouse retina (Fig. 3*D*,*D’*,*D’’*). As the faint stratification of *Tac1+* cells in the upper IPL strata resembled the distinct pattern for dopaminergic AC stratification, we performed TH antibody staining to test the possibility of dopaminergic cells being marked by this line, but that did not appear to be the case (Fig. 3*E*,*E’*,*E’’*).

**Figure 4.**
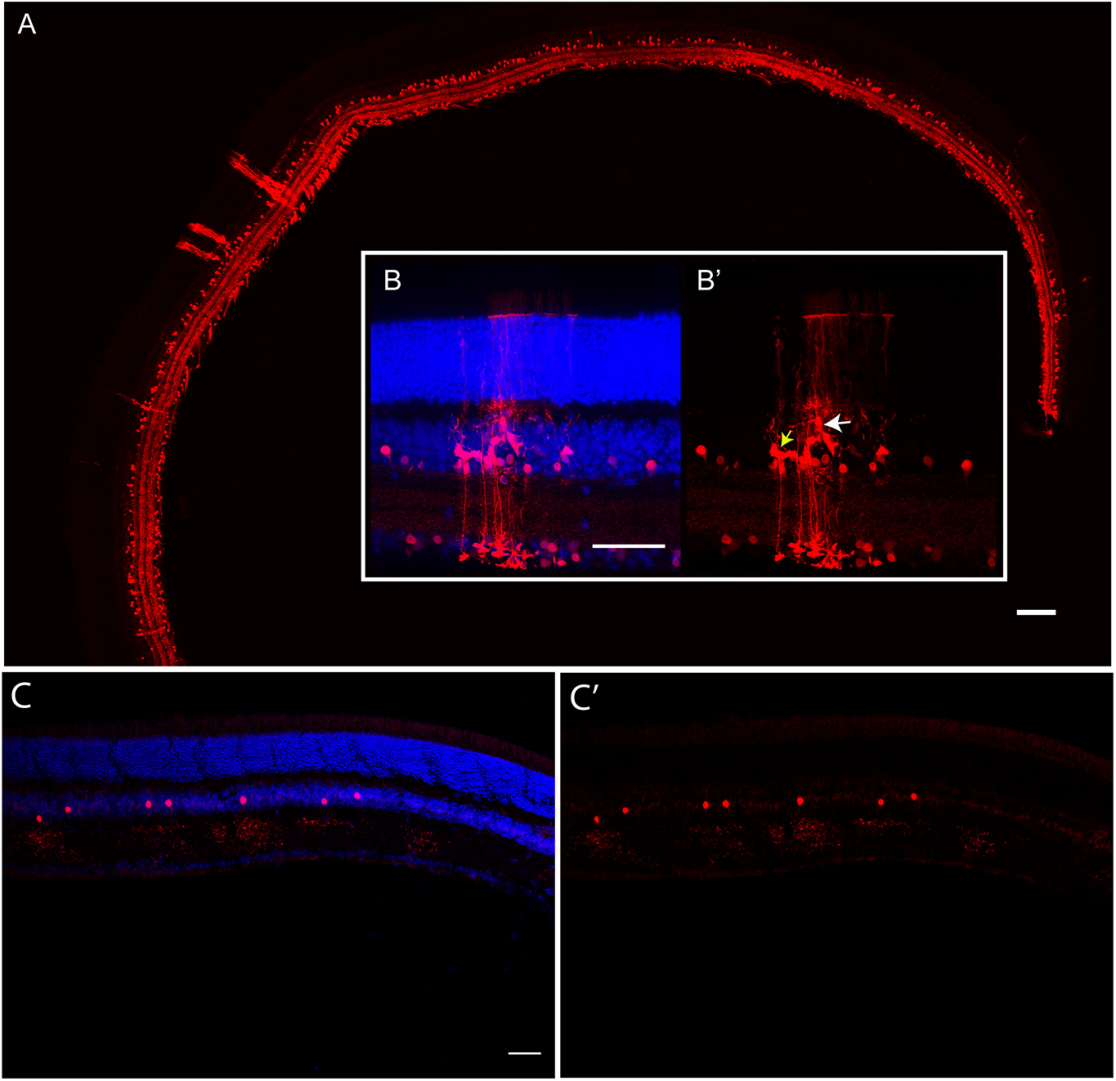
Global reporter expression in *Tac1::IRES-cre* and *Camk2a-cre* mouse lines. ***A***, While the majority of cells marked with a Tac1-Cre expression history were ACs, occasional clusters of Mueller glia were observed in cross-sections of the retina, in addition to AC and RGC subtypes. ***B***, ***B’***, Upon closer inspection, multiple tdTom+ Mueller glia (yellow arrow) and a few BPCs (white arrow) were visible (40×). Amongst the Cre-lines we have tested for retinal expression history, the *Tac1::IRES-cre* line was the only one with these clusters of different cell types (including Mueller glia) in a few locations, and there was a lack of them elsewhere in the retinal cross-sections. ***C***, ***C’***, Sparse tdTom+ ACs were present in the *Camk2a-cre* reporter mouse retina and there was a lack of labeling in other cell types. White scale bars: 50 μm.

Examination of the entirety of the *Tac1::IRES-cre-tdTom* sections at low magnification (Fig. 4*A*) also showed the distinct three-lamina stratification pattern of tdTom+ processes. In addition, we observed a few red Mueller glia with their distinctive morphology, spanning the entire radial dimension of the retina in several sections, but not all. The tdTom+ Mueller glia were observed to localize to a few discrete areas in the entirety of the retina. A closer look at a densely red area confirmed the presence of Mueller glia (Fig. 4*B*).

### *Camk2a-Cre* line fate maps to a unique AC type

**Figure 5.**
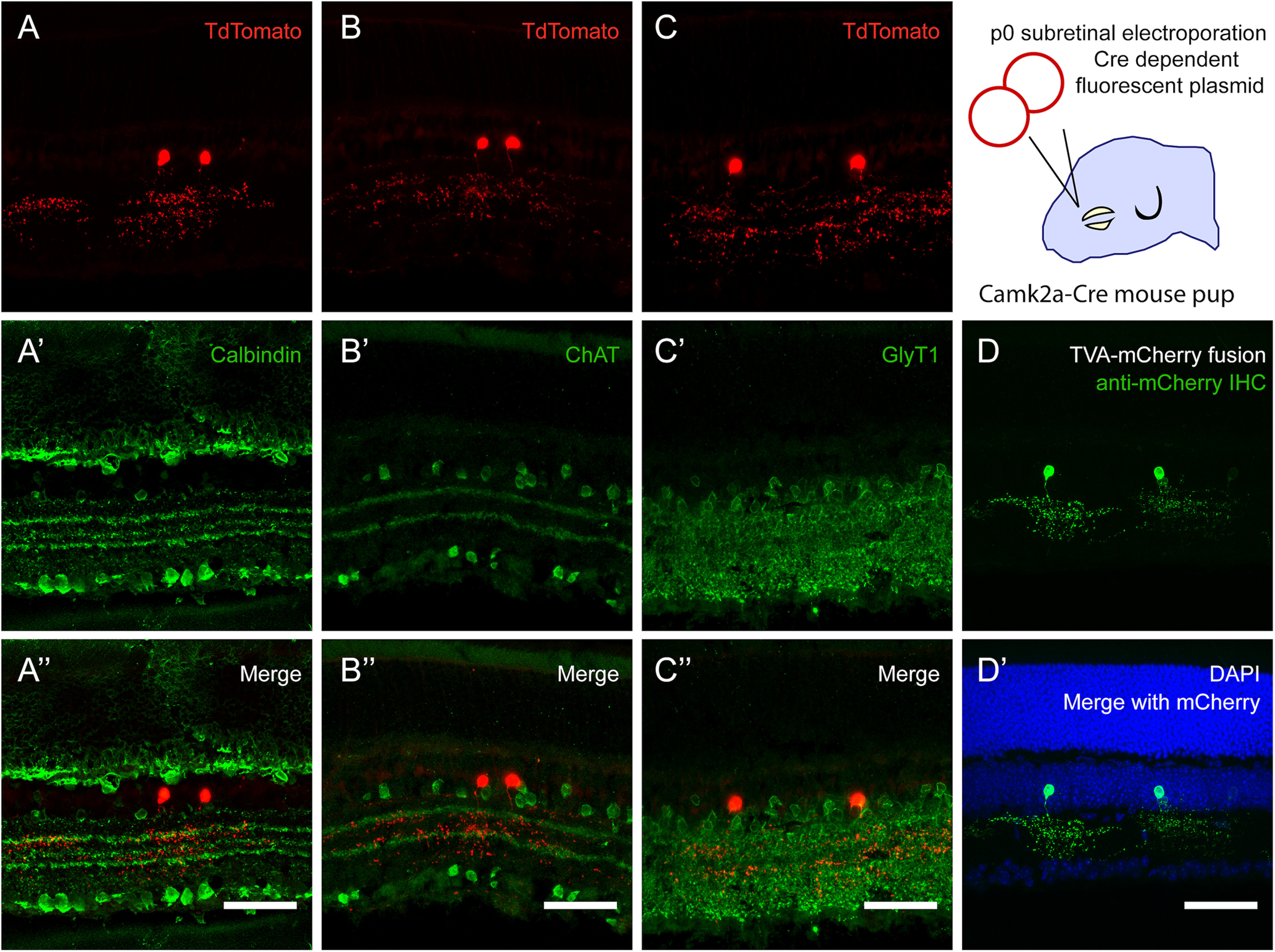
*Camk2a-cre* line fate maps to an AC type with unique morphology. ***A***, ***B***, *Camk2a-cre* AC did not express ChAT or calbindin (no yellow cells). However, the ChAT and calbindin bands collectively revealed that the medium sized dendritic arbors of the *Camk2a-cre* AC stratified mostly between levels 2 and 4, and excluded layers 1 and 5. ***C***, The ACs labeled by Camk2a-Cre expression history were glycinergic (Glyt1+, tdTom+ yellow cells). ***D***, Unique morphology of the AC marked by the Camk2a-Cre line could be visualized more clearly by the delivery of a Cre-dependent fluorescent plasmid into the subretinal space of mouse pups. Although the AC arbor appears uniformly bushy at a first glance with the TdTomato reporter, it seemed to have three distinct layers of stratification in the IPL, corresponding roughly to layers 2, 3, and 4. White scale bars: 50 μm.

CAMK2 is a calcium-activated serine/threonine kinase that is abundantly expressed in the brain and carries out multiple learning/memory related functions (Chia et al., 2018). *Camk2a* is known to encode for a subunit of CAMK2. Unlike the other Cre lines examined, preliminary evaluation of the *Camk2a-cre-tdTom* retinal sections in the presence of DAPI revealed red cell bodies only in the INL (Fig. 2*B*). tdTom+, sparsely labeled INL cells, which were located in the inner part of the INL, where AC somas are located. The fluorescent reporter intensity appeared fairly uniform among the labeled cell bodies (Fig. 4*C*,*C’*). The red processes spanned several strata in the middle of the IPL, yet they were diffuse and appeared to mark a distinct morphologic AC type. More specifically, the dendritic processes of these medium-arbor cells were diffusely distributed at the boundary of the a and b sublaminae of the IPL. IHC indicated that the dendritic arborization was particularly dense right over the upper calbindin band (Fig. 5*A*,*A’*,*A’’*, green) and between the two ChAT bands (Fig. 5*B*,*B’*,*B’’*, green) in the IPL. Furthermore, the ACs with Camk2a-Cre expression history (red) were not co-labeled with anti-ChAT or anti-calbindin. The cell bodies of *Camk2a-cre-tdTom ACs* (referred to as Camk2a-ACs from here on) were located consistently above the calbindin-positive ACs (Fig. 5*A’’*,*B’’*). To examine the neurotransmitter content of the Camk2a-ACs, IHC for GlyT1 immunostaining was used. The ACs with Camk2a-Cre recombination (red) were clearly glycinergic (green), as expected from their general morphologic appearance, as glycinergic ACs typically have diffuse, narrow or medium sized arbors (Fig. 5*C*,*C’*,*C’’*).

In order to visualize the dendritic arbors of the individual Camk2a-ACs more clearly, an electroporation strategy was employed. A Cre-sensitive reporter plasmid was electroporated at an early stage of development (e.g., at p0) subretinally into the eyes of *Camk2a-cre* mouse pups. This strategy can provide a more complete and sparse labeling of neuronal processes, as compared with the reporter mouse approach. With the *Camk2a-cre* line, the electroporations revealed the bistratified nature of the dendritic arbor more prominently, along with an asymmetrical OFF arbor. Consequently, we were able to match the Camk2a-AC morphology to one of the fairly common medium-field AC types in the Helmstaedter et al. (2013), serial block-face scanning electron microscopy (SBEM) atlas: type 42 or AC38–56.

### The *Scx-cre* mouse line has Cre-mediated recombination in multiple AC types

SCX is a basic helix-loop-helix transcription factor, critical for early tendon formation (Schweitzer et al., 2001). There has been no previous report of SCX expression in the retina, yet we became interested in the *Scx-cre* mouse line because of a scRNA profiling dataset (Trimarchi et al., 2008). This single-cell database indicated endogenous *Scx* expression in the mouse retina, including in some but not all retinal progenitor cells from embryonic day (E)14 to E16, and in a subset of ACs and BPs in the neonatal retina. *Scx* expression does not seem to persist in the postnatal retina as no expression could be detected via RT-PCR in p21 mouse retina, whereas p0 and p10 retinas both demonstrated Scx expression (data not shown).

Sections from the *Scx-cre-tdTom* mice were counterstained for DAPI, and red cell bodies with Scx-Cre expression history could be observed in the outer nuclear layer (ONL), INL, and GCL. A few faintly and sparsely labeled cells were also seen in the ONL (Fig. 2*C*), which appeared to be rod PRs, since they stained negative for peanut agglutinin (PNA), a cone PR marker (data not shown). The majority of the red somas were found in the INL and these cells evidently had the brightest tdTom expression of all the retinal cells labeled in *Scx-cre-tdTom* retinae.

**Figure 6.**
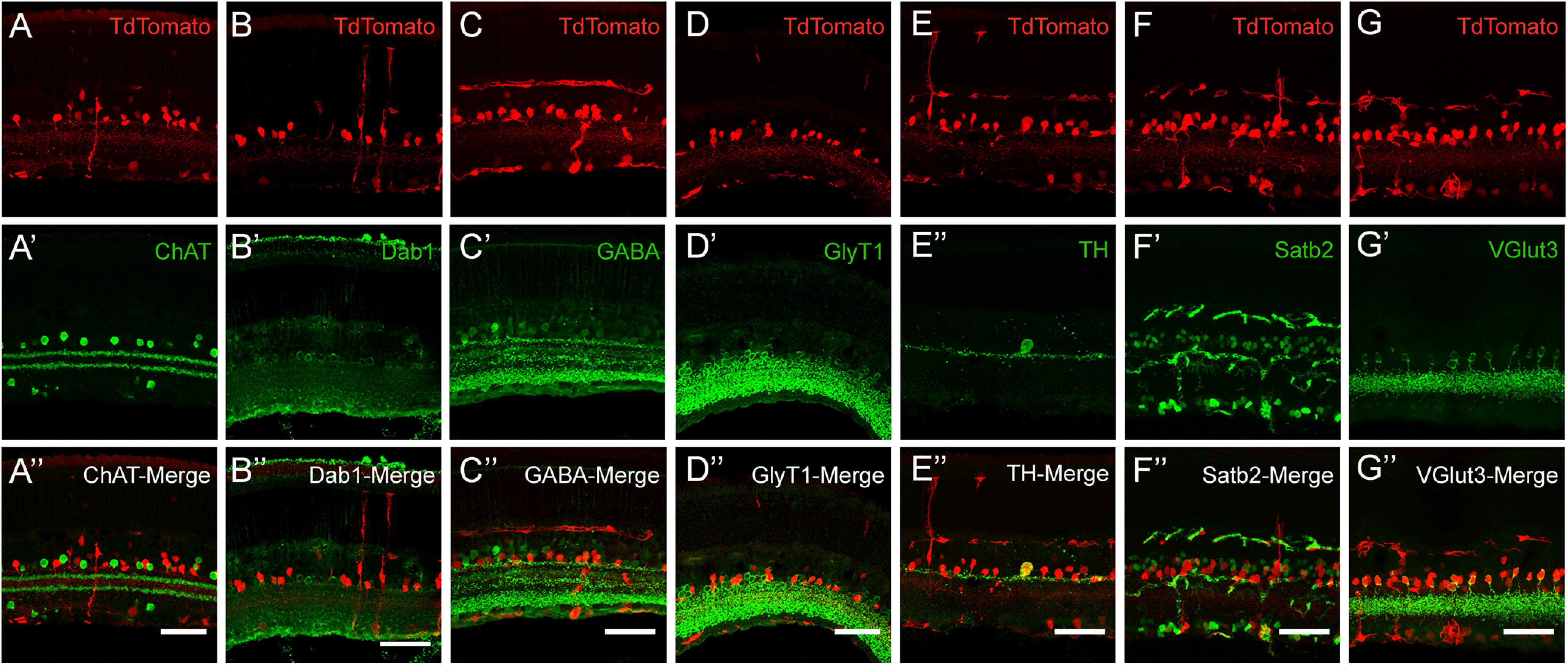
*Scx-cre* fate maps to multiple AC subtypes, including the Vglut3+ subtype. *Scx-cre* mouse line Cre expression history was assayed for all major AC subtypes, including ChAT (cholinergic ACs), Dab-1 (AII ACs), GABA (GABAergic ACs), GlyT1 (glycinergic ACs), TH (dopaminergic ACs), Satb2 (non-glycinergic, non-GABAergic ACs), Vglut3 (Vglut3+ ACs). ***A–C***, No ChAT+, Dab-1+, or GABA+ ACs were tdTom+ (red), indicating the absence of these markers in the Scx-Cre fate mapped population. ***D–F***, A subset of GlyT1+, TH+, and Satb2+ ACs were tdTom+ (red). ***G***, All Vglut3+ ACs were tdTom+ (red). White scale bars: 50 μm.

The positioning of the red cell bodies among the INL cells (close to the IPL) were consistent with the majority of these cells being ACs. A small number of Mueller glia and BP cells were also evident, as judged by their characteristic morphologies and locations. The majority of the IPL stratification was restricted to two sublaminae (laminae 1 and 3) in the IPL, the more prominent one being in the outer part of the IPL. To further investigate the subtype identities of ACs with *Scx-cre* history (referred to as Scx-ACs from here onward), we conducted IHC with a range of AC markers. Most striking of all, 100% of glutamatergic (VGlut3-positive) ACs had *Scx-cre;tdTom* history, comprising about a third of total ACs mapped by this mouse line. This demonstrated a significant skew toward VGlut3-ACs since as previously reported, Vglut3^+^ ACs only accounted for 4% of total ACs in the mouse retina (Voinescu et al., 2009). In addition, examination of other neurotransmitter content showed that a number of Scx-ACs were positive for the glycinergic marker GlyT1 (Fig. 6*D*,*D’*,*D’’*), overlapped with ∼50% of dopaminergic (TH-positive) ACs (Fig. 6*E*,*E’*,*E’’*). We also noted the presence of Satb2, a non-glycinergic non-GABAergic AC marker, in a few Scx-ACs (Fig. 6*F*,*F’*,*F’’*). However, the Scx-ACs were not GABAergic (Fig. 6*C*,*C’*,*C’’*, GABA in green), and had no overlap with ChAT-positive starburst ACs nor Dab1-positive AII cells (Fig. 6*A*,*A’*,*A’’*, ChAT in green, *B*,*B’*,*B’’*, Dab1 in green).

For cells with red somas located in the GCL, co-staining for the Brn3a antibody, an RGC marker, in *Scx-cre-tdTom* retinal sections revealed tdTom fluorescence in a number of RGCs. There were also several Brn3a negative red somas in the GCL (data not shown), which could be displaced ACs.

## Discussion

ACs are the most diverse neuron type in the retina and they are arguably the least understood in terms of their contributions to visual processing. Over the last decade, considerable progress has been made toward decoding neuronal diversity at the anatomic and molecular levels. The mammalian retina has served as an excellent system toward that goal because of its accessibility and the relatively small number of neuronal cell types as compared with the brain. First, a SBEM reconstruction of the mouse inner retina revealed 45 types of ACs, followed by the original scRNA-seq platform identifying 21 transcriptionally distinct AC clusters (Helmstaedter et al., 2013; Macosko et al., 2015). Both efforts were followed by many others studying neuronal diversity in the retina, including variations of scRNA-seq studies of the human retina. Some of these findings were limited in their capacity to globally profile ACs and RGCs because of the fact that only a small number of these types could be obtained with the available preparation techniques, as compared with the overwhelming number of PRs (Lukowski et al., 2019; Menon et al., 2019). Most recently, a transcriptomics study focused on a thorough AC categorization and indicated 63 AC types in the mouse retina (Yan et al., 2020). These large-scale screening studies emphasize the need for complementary genetic tools that will enable access to individual AC types, or a few AC cell types, for definitive studies focused on their retinal function(s) (Vlasits et al., 2019). To achieve this level of insight, we need the ability to label, manipulate and ablate specific cell types. Applying site-specific recombinase technologies using mouse genetics remains an optimal method of accessing specific cell types *in vivo*.

We have chosen to characterize lines employing the Cre-lox system for accessing small subsets of ACs in the retina. The use of Cre lines have been instrumental in understanding retinal function. Several studies published in the last decade identified Cre lines valuable to neuroscientists for accessing neuronal subsets in the retina, such as RGCs or ACs (Martersteck et al., 2017; Jo et al., 2018). In the current study, we were able to identify three Cre mouse lines with unique AC expression patterns in the mouse retina. First, we examined the Cre reporters for fluorescent labeling density/intensity and soma locations. Three out of three lines we tested had Cre-mediated recombination in AC subsets, which were then surveyed for morphologic identifiers. With the exception of the *Tac1::IRES-cre* line, where occasional Mueller glia “islands” were observed, the tdTom+ cell distribution was fairly uniform across the retina in all mouse lines. IPL stratification is critical for synaptic connectivity and is used as a criterion for identification of interneurons and RGCs (Sümbül et al., 2014). Two of the tested Cre lines (*Tac1::IRES-cre* and *Camk2a-cre*) marked neurons sparsely enough that we could identify stratification patterns in the IPL. This suggests that they are particularly promising resources for further studies of visual function. None of the Cre lines we have employed in this study demonstrated any gross changes to retinal organization.

We were initially interested in the *Tac1::IRES-cre* line because of some previous work using microarrays and single ACs. *Tac1* expression was found in a small GABAergic AC population, potentially in a unique AC type (Cherry et al., 2009). Indeed, we report here that the *Tac1-cre* line marked mostly GABAergic ACs, and a small number of RGCs in the mouse retina. We also find that our observation is consistent with the report of Yan and colleagues, who identified six transcriptionally-distinct and GABAergic cell clusters among seven *Tac1-high* AC populations in the mouse retina (Yan et al., 2020). *Tac1-cre* history in the retina was briefly described elsewhere as “positive in ACs and RGCs” but no further details were provided (Martersteck et al., 2017). We observed that *Tac1-tdTom* processes in the IPL were complimentary to ChAT-positive starburst AC processes in their stratification patterns. The significance of this needs to be further investigated. However, based on these findings we speculate that the connectivity patterns of Tac1-ACs are quite distinct from the well-studied direction selective circuit in the mammalian retina, which utilizes ChAT ACs. Overall, *Tac1-cre* line should be of utility as a marker of specific AC subsets. Moreover, it can be combined with *flp* lines or plasmid electroporations in the mouse retina for an intersectional strategy that could more narrowly specify cell types, similar to previous examples of intersectional genetic approaches (Jo et al., 2018).

Along those lines, it can be argued that it is a rare and useful find when a Cre mouse line targets a single neuronal cell type, as in the case of the *Camk2a-Cre* line. The Camk2a-AC is a possible match to a specific medium-field AC described in Helmstaedter et al. (2013), SBEM database, the AC38–56. As assessed by stratification patterns and overall morphologic appearance, the AC38–56, might correspond to the A8 cell in the literature (Lee et al., 2015). However, we have seen little resemblance between the reported morphology of the mouse A8 (Lee et al., 2015; Yadav et al., 2019) and the ACs with *Camk2a* expression history. The Camk2a-ACs, as well as the AC38–56, are bistratified. For the AC38–56, as the name implies, stratification depth peaks at ∼38% and 56% of the IPL. The Camk2a-ACs elaborate processes that are particularly dense between and below the ChAT bands, with remarkably bushy processes in the ON layer. In contrast, the mouse A8 processes appear restricted to two distinct layers. It will be intriguing to study the *Camk2a-Cre* line in the future with a focus on this AC’s physiological properties and connectivity patterns, without the confounding factor of other neuronal cell types.

Camk2 isoforms are widely expressed in the retina and recently have been found to mediate the angiogenic actions of a range of growth factors in human retinal endothelial cells (Ashraf et al., 2019). CAM2KA, in particular, is an enzyme known to induce memory formation at chemical synapses and has been described to potentiate electrical coupling in the retina (Tetenborg et al., 2019). According to Tetenborg et al. (2017), *Camk2a* is strongly expressed in starburst ACs, and in a few other cell types (Tetenborg et al., 2017). The pattern of labeling seen here for *Camk2a-cre* thus does not reflect the endogenous pattern of expression of Camk2a, which is often the case for transgenic mouse lines. Similarly, not all transgenic reporter lines report in the same way. Even when the reporter line uses a broadly active promoter, such as CAG in the Rosa26 locus, differences have been observed in the expression patterns of the reporter when crossed to the same Cre line (Madisen et al., 2010). In the particular case of *Camk2a-cre*, Ivanova and colleagues found that the *Camk2a-cre* line crossed to a floxed EYFP reporter yielded a weak and sparse expression in BPs, ACs, and RGCs. A further definition of these cell types was not conducted (Ivanova et al., 2010). The different labeling pattern that we observed could be attributed to the different reporter lines employed. In our case, we were able to visualize the same morphologic AC type with two different methods (Ai9 Cre-dependent reporter cross and reporter plasmid electroporations). Hence, it is vital to check expression patterns with the intended Cre reporter line and/or use complementary techniques to study Cre activity. Furthermore, our finding that the Camk2a-ACs can be electroporated at birth indicates that these neurons continue to emerge postnatally, as electroporation tends to target mitotic cells and their newly postmitotic daughters (Matsuda and Cepko, 2004). This is consistent with the observation that the glycinergic ACs are late born in the mammalian retina and provides another way of accessing/manipulating these cells.

ACs characteristically use either one of the two inhibitory neurotransmitters: glycine or GABA and are thus categorized as glycinergic or GABAergic (Pourcho and Goebel, 1983; Menger et al., 1998) We have found that the Tac1-ACs are largely GABAergic whereas the Camk2a-ACs are glycinergic, consistent with the morphologic descriptions in the literature. There is a third category: ACs with very low expression (or no expression) of glycine or GABA, essentially with unknown neurotransmitter content (Kay et al., 2011). Although their functional role in the mammalian retina remains unknown, Satb2 has been used as a marker for this population. We have tested it in our *Scx-cre* line, and it appeared heterogenous as it marks a subset of Scx-ACs. Many ACs release one other neurotransmitter or neuropeptide, in addition to GABA or glycine, such as dopamine, somatostatin, glutamate, and NPY. It is thought that this feature might enable ACs to carry out multiple roles in visual processing (Diamond, 2017). In light of a recent study where neuropeptide expression has been assigned to more specific AC populations (Yan et al., 2020), a more thorough analysis of AC markers would be necessary to examine the Cre lines reported here.

SCX is a bHLH transcription factor primarily expressed by tendon and ligament cells. Previous studies have shown that *Scx* is necessary for connective tissue development (Blitz et al., 2009; Chen and Galloway, 2014). The *Scx-cre* mouse line is primarily used in the study of tendon/ligament biology and has been shown to recombine in the nervous system (Lim et al., 2017; Luo et al., 2020); however, to our knowledge, this is the first study indicating Scx-Cre mediated recombination in retinal cells. Notably, we have observed that Scx-ACs are heterogenous. This population includes the entirety of glutamatergic ACs, as well as about half of the dopaminergic ACs, Satb2+, ACs and displaced ACs. We conclude that *Scx-cre* line is not suitable for specific targeting of AC subpopulations because of its heterogeneity. However, it can be used in an intersectional manner, in combination with *flp* lines, plasmid electroporations or other gene delivery strategies, to tease apart and manipulate subsets of ACs, as indicated in its description of Cre expression history in this study. It will also be important to determine how the retinal expression of the newly generated Cre mouse lines using the same promoters/knock-in strategies reported in this manuscript, such as the *Scx-cre* by Yoshimoto and colleagues, will compare to our retinal expression data (Yoshimoto et al., 2017). We have previously observed major differences in Cre expression history in the retinae of mouse lines using the same promoter elements (Crh-Cre line, data not shown, summarized in Table 3).

Overall, there are multiple benefits to using Cre-expressing mice to study the nervous system. They can be used to delete gene targets, turn on optogenetic genes, enable expression studies for drugs or calcium indicators, and ablate specific cell types (Brockschnieder et al., 2004; Zeng and Madisen, 2012). Furthermore, Cre lines can be used to drive receptor expression for connectivity analysis with viral targeting and tracing (Seidler et al., 2008; Beier et al., 2013). Finally, researchers are not limited to floxed mice for Cre specific manipulations. Several other techniques can be applied to achieve Cre driven expression, such as delivery of Cre dependent viruses (e.g., floxed AAVs) and plasmids (Haery et al., 2019). The retina is an easily accessed part of the nervous system with a singular sensory input (light), enabling genetic manipulations as well as physiological assays. ACs are a particularly intriguing set of inhibitory neurons in the retina because of their extreme morphologic and functional diversity. They play roles as important and unique as contributing to directional selectivity, night vision, and even diseases, such as congenital nystagmus when they are dysfunctional (Yonehara et al., 2016). In the near future, with the availability of more genetic tools that give us access to individual AC types, we will continue to learn more about their functions in visual processing.

## Synthesis

Reviewing Editor: Siegrid Löwel, University of Goettingen Decisions are customarily a result of the Reviewing Editor and the peer reviewers coming together and discussing their recommendations until a consensus is reached. When revisions are invited, a fact-based synthesis statement explaining their decision and outlining what is needed to prepare a revision will be listed below. The following reviewer(s) agreed to reveal their identity: Andreas Feigenspan.

The authors use four existing Cre-expressing transgenic mouse lines to target various amacrine cell types, the most diverse cell type in the mammalian retina. Since the roles of the majority of amacrine cells in retinal signal processing is still poorly understood, a thorough analysis of the mouse lines presented in this manuscript may serve as a valuable tool in the future study of amacrine cell function. At the moment, however, the manuscript is mainly descriptive with e.g. no clear suggestion for the use of these mouse lines.

Amacrine cells represent the most diverse cell class in the mammalian retina: recently, 63 different types have been identified based on single-cell RNAseq in the mouse retina (Yan et al., 2020). However, less than 10 types have been studied in detail yet. One reason for this is that amacrine cells are hard to target reliably. Cre-expressing mouse lines may provide good tools to enhance the accessibility of amacrine cells. Here, this paper sets in. The authors screened for mouse lines expressing Cre recombinase in amacrine cells. They identified four different mouse lines and investigated reporter gene expression together with neurotransmitter and cell marker expression. Only one of these lines labels a morphologically distinct amacrine cell and therefore provides a useful tool to study this cell type (which seems to be largely unknown). The other mouse lines, however, show broader Cre expression in retinal neurons and sometimes even glia cells, making the use of these mouse lines less straightforward.

In summary, we believe that a more precise characterization of amacrine cell types as well as a judgement of potential developmental defects in the transgenic mouse lines would increase the scientific value of the present study a lot. Furthermore, quantifications are presently absent although it would have been interesting to see whether reporter expression varies between individual mice.

More detailled comments:

1. The retina sections sometimes have a low quality (see Figure 2B) and retinas are not aligned. Also, some stainings look awkward, e.g., the Brn3a staining shown in Figure 7B.

2. Figure 1 might serve as a broad overview, which is probably intended, but it is appears slightly oversimplified. In particular, very little information is provided on cell types and connectivity in the mammalian retina.

3. In Figure 2, section quality for Camk2a-cre and VGAT::IRES-cre is worse than for the other two mouse lines. Especially, the lack of most of the ONL in the VGAT::IRES-cre line is puzzling. In addition, the DAPI staining is overexposed. In general, are any changes of retinal organization known for the transgenic lines used in this study, which might affect the distribution and expression patterns of amacrine cells? This should at least be mentioned in the discussion.

4. Unfortunately, no attempts were made to assess cell densities to estimate whether all cells of a given type are labeled or not. This represents a useful piece of information for other researchers as it greatly determines the suitability of the mouse line for physiological studies.

5. Related to this: Figure 4 at least, allows for one mouse line to judge the retinal distribution of reporter gene expression. Similar images should be provided for the other lines as well.

6. Lines 29/23: This is true only for the photopic pathway.

7. Line 211: This is difficult to assess from the stainings in Figure 3B. It seems that the overlap with GABA is modest. The same holds true for the colocalization with GlyT. Given that the majority of amacrine cells are either GABAergic or glycinergic, the combination of GABA and GlyT staining shown here might not fully represent this population.

8. Figure 3E: This staining does not show a distinct cell body. Could you provide another section with a more well-defined somatic labeling?

9. Line 266 and Figure 2C: These faintly labeled cells in the ONL are not visible in Fig. 2C, at least not at this resolution and staining intensity.

10. Line 282 and Figure 6D: Can you discriminate between type 1 and type 2 TH-positive amacrine cells. Shown in Fig. 6D is a type 1 cell. Does 50% overlap refer only to type 1 cells or to both DA cell types?

11. Line 394 and Figures 3 and 7: Brn3a stainings in Fig. 3 and 7 look rather different. Do the authors have any explanation for this discrepancy?

## Author Response

We thank you again for your constructive feedback. Below you can find our itemized responses to each comment. Edits to the original manuscript are highlighted in yellow in the resubmitted manuscript.

1. The retina sections sometimes have a low quality (see Figure 2B) and retinas are not aligned. Also, some stainings look awkward, e.g., the Brn3a staining shown in Figure 7B.

We agree with this statement and have replaced Figure 2B with a new one. However, we no longer maintain the VGAT::IRES-cre mouse line and unfortunately do not have better images to reflect the Brn3a staining. We have therefore chosen to remove the VGAT line from the manuscript. (See below for details)

2. Figure 1 might serve as a broad overview, which is probably intended, but it is appears slightly oversimplified. In particular, very little information is provided on cell types and connectivity in the mammalian retina.

Figure 1 has been expanded to provide more information on cell types and connectivity. Specifically, a typical cone circuit and a few different morphological types of ACs were highlighted to reflect the diversity in AC signaling.

3. In Figure 2, section quality for Camk2a-cre and VGAT::IRES-cre is worse than for the other two mouse lines. Especially, the lack of most of the ONL in the VGAT::IRES-cre line is puzzling. In addition, the DAPI staining is overexposed. In general, are any changes of retinal organization known for the transgenic lines used in this study, which might affect the distribution and expression patterns of amacrine cells? This should at least be mentioned in the discussion.

We agree with the reviewer, and have replaced the Camk2a-cre image with a new one where section quality and exposure are considerably improved. We also indicated that there were no changes to the retinal organization in the Discussion (Lines 325-327) We unfortunately do not have alternative images to address the comments about the VGAT::IRES-cre line. Therefore, we have removed this mouse line from the manuscript, as indicated earlier in the response to comment #1.

4. Unfortunately, no attempts were made to assess cell densities to estimate whether all cells of a given type are labeled or not. This represents a useful piece of information for other researchers as it greatly determines the suitability of the mouse line for physiological studies.

We agree with this statement, but unfortunately, we no longer maintain these mouse lines in our colony so we were unable to document additional assessments.

5. Related to this: Figure 4 at least, allows for one mouse line to judge the retinal distribution of reporter gene expression. Similar images should be provided for the other lines as well.

We completely agree that retinal distribution is a very useful piece of information. To this end, we have provided an additional low magnification (10x) image of the Camk2aCre-TdTom reporter mouse in Figure 4C and C’. We have also added a statement to Discussion indicating that the tdTom+ cell distribution was fairly uniform for mouse strains other than Tac1Cre, with the Camk2a line tdTom+ cell distribution being considerably sparser than the others. (lines 319-321).

6. Lines 29/23: This is true only for the photopic pathway.

Thank you for the reminder, this section has now been revised (Line 30-32).

7. Line 211: This is difficult to assess from the stainings in Figure 3B. It seems that the overlap with GABA is modest. The same holds true for the colocalization with GlyT. Given that the majority of amacrine cells are either GABAergic or glycinergic, the combination of GABA and GlyT staining shown here might not fully represent this population.

This section was revised to reflect a clearer description of the observations regarding the GABA and glycine expression. We are unable to provide additional images. However, in the revised Discussion (line:332-334), we note that our observations are consistent with another report in the literature: “We also find that our observation is consistent with the report of Yan, Laboulaye et al., who identified 6 transcriptionally-distinct and GABAergic cell clusters among 7 Tac1-high AC populations in the mouse retina.”

8. Figure 3E: This staining does not show a distinct cell body. Could you provide another section with a more well-defined somatic labeling?

We unfortunately do not have an additional section with a more distinct cell body. We appreciate the reviewer’s concern and can remove this panel from the figure should the reviewers and editor recommend so.

9. Line 266 and Figure 2C: These faintly labeled cells in the ONL are not visible in Fig. 2C, at least not at this resolution and staining intensity.

Thank you for this feedback: We have increased the brightness and contrast of the existing panel 2C and we believe cells are now clearly labeled in the ONL.

10. Line 282 and Figure 6D: Can you discriminate between type 1 and type 2 TH-positive amacrine cells. Shown in Fig. 6D is a type 1 cell. Does 50% overlap refer only to type 1 cells or to both DA cell types?

We agree that the Fig. 6D is showing a type 1 cell. Unfortunately, we were not able to discriminate between the two TH types with the number of images we have at hand and the overlap suggests an overall TH-positivity without this discrimination.

11. Line 394 and Figures 3 and 7: Brn3a stainings in Fig. 3 and 7 look rather different. Do the authors have any explanation for this discrepancy?

We agree that the Brn3a staining in Fig. 7 looks rather different than Fig. 3, and in not direct alignment with the literature. As we could not explain this difference with the images and slides at hand, we have chosen to remove Fig. 7 from the manuscript.
